# Improved Prediction of Eurasian Beaver Gnawing Preferences in Riparian Habitats: A Machine Learning Approach

**DOI:** 10.1002/ece3.72649

**Published:** 2025-12-17

**Authors:** Giovanni Trentanovi, Emanuele Santi, Emiliano Mori, Andrea Viviano, Alessio Giovannelli, Maria Laura Traversi, Francesco Sarnari

**Affiliations:** ^1^ Research Institute on Terrestrial Ecosystems—National Research Council (IRET‐CNR) Sesto Fiorentino FI Italy; ^2^ Institute of Applied Physics—National Research Council (IFAC‐CNR) Firenze Italy; ^3^ National Biodiversity Future Center (NBFC) Palermo PA Italy; ^4^ Istitute of Bio‐Imaging and Complex Biological Systems—National Research Council (IBSBC‐CNR) Cefalù PA Italy

**Keywords:** animal behavior, *Castor fiber*, deep learning, model comparison, random forest, riparian woodlands

## Abstract

In this study, we investigated the impact of Eurasian beavers ( *Castor fiber* Linnaeus, 1758) on riparian woodlands in Central Italy using Machine Learning (ML) techniques. Beavers are ecosystem engineers who may modify riverine ecosystems through dam building and foraging activities. Their gnawing activity can significantly alter the composition and structure of riparian forests. Traditionally, statistical models have been used to understand factors influencing beaver activity. Thus, this study explores the potential of ML algorithms for this purpose. We implemented three ML algorithms—Artificial Neural Networks (ANN), Support Vector Machines (SVM), and Random Forests (RF)—to analyze data collected from three Italian rivers. Data included in situ measurements of trees (diameter, distance from riverbank, species) and information on beaver damage (i.e., signs of gnawing activity and the type of impact on the single stem). A two‐step implementation has been proposed to predict whether a tree would be damaged by beavers and, if so, the severity of the damage (“low” or “high”). In the first step, three algorithms achieved high accuracy (up to 93% of damaged/undamaged trees correctly classified) and kept satisfactory performances even when trained with small subsets of the data (85% accuracy when trained with 20% of the data). In the second step, implemented over the subset of trees classified as damaged to identify those with low or high damage severity classes, the algorithms reached accuracy (85%) comparable to Step 1, despite the smaller subset available (159 samples out of 476 in the total dataset). This suggests that ML could significantly reduce the amount of field data collection needed to assess beaver impacts. The results of the analysis also suggested RF as the most suitable ML method for this kind of application in terms of both accuracy and computational cost. Moreover, the following key factors influencing beaver gnawing activity were identified: tree diameter and distance from the riverbank were the most important predictors, while tree species and site location had less influence. In summary, this study showed the potential of ML for analyzing beaver‐woodland interactions with a more effective and cost‐efficient sampling effort and better understanding the main factor influencing beaver gnawing activity. Future research should test the dataset from different geographical ranges, as well as incorporating data on long‐term foraging sites.

## Graphical abstract

Rapid assessment for ecosystems impact assessment is necessary to implement timely and effective ecosystem management strategies. Machine Learning (ML) can help to guide new collection data protocols focused on less but effective samples that can be used to predict rodents' impacts on riverine ecosystems.

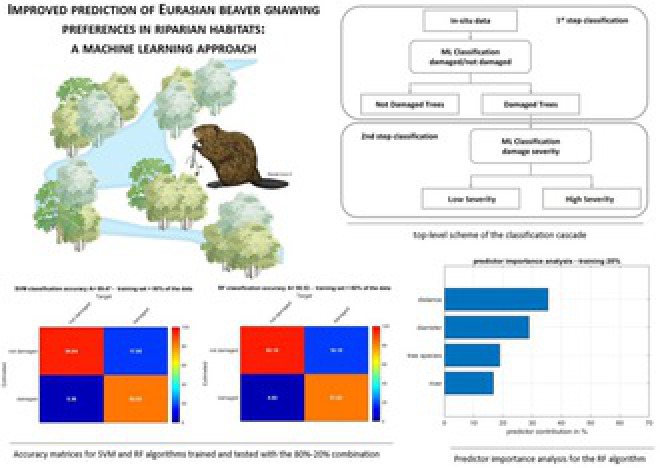

## 1 Introduction

### 1.1 Eurasian Beaver Presence in EU, Behavior, and Impacts on Riparian Forests

**FIGURE 1:**
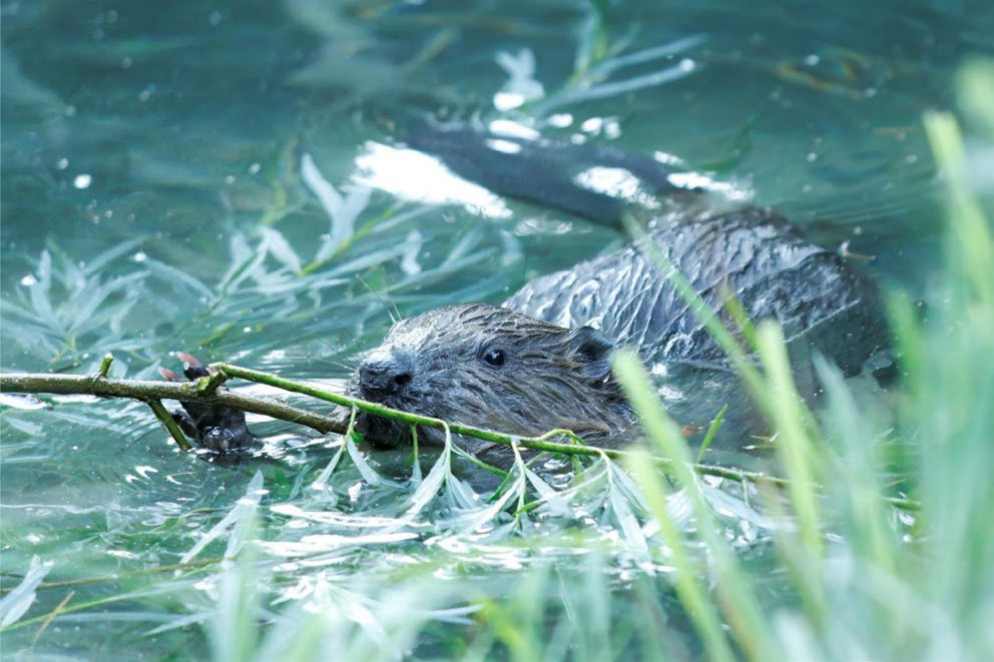
Eurasian beaver carrying white willow twigs for dam construction in the Slizza river (Tarvisio, Italy). Credits by Renato Pontarini.

The Eurasian beaver ( *Castor fiber* Linnaeus, 1758) is a large rodent (Figure 1) capable of inhabiting and establishing itself in a wide range of environments, from natural habitats to highly anthropized landscapes (Campbell‐Palmer et al. 2021).

At the beginning of 1900, the range of the Eurasian beaver was limited to a few small‐sized refugia between France and Mongolia, hosting fewer than 1200 individuals (Halley and Rosell 2002). Currently, the Eurasian beaver shows reproductive populations in most of its original range, from Western Spain to Mongolia (Halley et al. 2021; Kodzhabashev et al. 2021; Calderón et al. 2022; Paladi and Cassir 2022; Mori et al. 2024). Beaver species are considered “ecosystem engineers,” as they may modify the riverine ecosystem where they live (Rosell et al. 2005) in a very short time period, with deep implications on riparian woodland habitats (Brazier et al. 2021). Woodland consumption of riparian forests is one of the main direct impacts of beaver activities and is due mainly to feeding (Nolet et al. 1994; Fustec et al. 2001) and river dam creation (Puttock et al. 2017; Brazier et al. 2021). Specifically, regarding the stand tree layer, some studies highlight the reduction of living biomass and the increasing deadwood quantity following beaver gnawing activity (Thompson et al. 2016). More canopy gaps are formed, increasing species selection and light availability, thus favoring the regeneration of pioneer species (Stringer and Gaywood 2016), as well as the development and growth of young trees (Gaywood 2015), with a significant effect on stand structure (Trentanovi et al. 2025). Beaver preferences for tree species are influenced by several factors, including taxonomy, shoot diameter, and distance from the riverbank, as well as interactions among these variables (Nolet et al. 1994; Campbell‐Palmer et al. 2016). For example, species such as willow (*Salix* spp.) and aspen (*Populus* spp.) are often preferred, but the relative importance of tree characteristics and location in shaping beaver gnawing activity remains debated (Haarberg and Rosell 2006; Zwolicki et al. 2019; Mahoney and Stella 2020).

### 1.2 Statistical Modeling on Wild Fauna, With a Focus on Beavers

The necessity of comprehending the influence of various factors on the impact of wild fauna on ecosystems has been addressed in numerous studies using classical statistical approaches (e.g., Seidl et al. 2011; Northrup et al. 2022). Several concerns have been raised about the performances of these models and techniques when considering the complex patterns of highly interactive environmental covariates (Brugere et al. 2023). Studies focused on beavers included likelihood ratio test (O'Connell et al. 2008), multiple logistic regression (Nolet et al. 1994), general linear model (Haarberg and Rosell 2006; Jackowiak et al. 2020), generalized linear mixed models (Juhász et al. 2022; Mikulka et al. 2022; Trentanovi et al. 2023), logistic models and spatial auto correlograms (Piton et al. 2020). The main limitations of regression models include the stringent preparatory statistical assumptions required for the data (Weiskittel et al. 2011), the quality and power of non‐parametric models in explaining phenomena (Hamidi et al. 2021), as well as the differences in sampling effort (whether this is spatial, temporal, or taxonomic), that can lead to improper results (Petersen et al. 2021). This latter issue is of particular interest in studies of wild fauna impact on natural ecosystems, as field data collection is often very time demanding and sampling effort is deeply influenced by sites' accessibility (e.g., for beavers: Zwolicki et al. 2019; Piton et al. 2020; Pejstrup et al. 2023). The species has a moderate reproductive rate, typically producing one litter of 2–6 kits per year, which is relatively high compared to other mammals of similar body size (Rosell and Campbell‐Palmer 2021), thus monitoring requires rapid information to obtain effective responses on site conditions and to implement related management actions (Casazza et al. 2023). Current literature emphasized the necessity of transcending statistical approaches and adopting novel technological tools that offer powerful means to interpret animal behavior (Couzin and Heins 2023). In recent years, the application of Machine Learning (ML) methods in forest ecology has grown rapidly, driven by the increasing computational capabilities of modern computers (Liu et al. 2018). ML algorithms learn patterns directly from data without requiring a priori assumptions, as traditional statistical methods do. This allows ML to handle redundant or noisy datasets and to address a wide variety of ecological problems with high accuracy (Mitchell 1997). ML has been successfully applied to species distribution prediction and habitat suitability modeling (e.g., Périé and de Blois 2016), carbon and energy flux estimation (e.g., Tramontana et al. 2015), hazard assessment (e.g., Zhang et al. 2023), stress physiology (Francini et al. 2023), and forest management (e.g., Silva et al. 2023). Despite these advantages, ML also has notable limitations. It typically requires large datasets for training, and there is a risk of exploiting apparent correlations rather than true causal relationships. Consequently, using ML as a “black box” without understanding the underlying ecological or physical processes is not recommended (Santi 2016). Beyond these well‐documented applications and limitations, several open research areas remain. One particularly relevant challenge is understanding wildlife biotic interactions using ML, which is still largely unexplored (Shoemaker et al. 2018; Tuia et al. 2022). For example, mammal reintroduction in rewilding forest ecosystems requires addressing multiple knowledge gaps, such as assessing ecological impacts and identifying strategic and tactical management options (e.g., Stringer and Gaywood 2016; Johnson et al. 2020).

This study aims to evaluate the potential of various Machine Learning (ML) methods (i.e., Support Vector Machines, Random Forests, and Artificial Neural Networks) for understanding the key factors influencing beaver gnawing activity on woody stems. Specifically, we examine how the amount of training data affects prediction accuracy. We hypothesize that the relative importance of environmental variables in forest riparian ecosystems impacted by beaver gnawing can be reliably assessed using robust, nonparametric ML techniques, with minimal assumptions about data structure.

## 2 Materials and Methods

### 2.1 Data Collection and Stand‐Level Structural Analysis

Data collection was carried out in summer and autumn of the years 2022 and 2023, within three rivers (Tevere, Merse, Ombrone) of Italy. Eurasian beavers started this area in 2019 (Mori et al. 2022), after an absence of more than 400 years. At this point, beavers can be said to be in their early‐ to middle‐expansion phase (Falaschi et al. 2024; Mori et al. 2024). The vegetation communities and the river flow regime are those typical of Mediterranean semi‐natural riparian areas. Specifically, two kinds of phytosociological alliances were surveyed, that is, *Populion albae* for Merse and Ombrone rivers and *Alnion glutinosae* for the Tevere river. We sampled 475 standing woody elements (including snags and stumps), distributed heterogeneously across six transects (plots 5 × 20 m) per river within two representative homogeneous riparian vegetation stands. Most of the beaver gnawing activity within the transect was observed before the year 2022. Indeed, since 2019 the beaver population has continued to grow, moving progressively to the southern river stands. Fresh signs were observed only occasionally across the entire forest stand, but always far from the surveyed plots. Dendrometric data were measured, and then structural parameters were calculated at the stand level. For living trees, we collected two diameters, one at 130 cm (i.e., diameter at breast height—DBH) and at 15 cm above the ground, as well as gnawing damage type and severity (Table 1). With the term “damage” we intend the presence of beaver gnawing activity signs on the single tree stem. For stumps (height < 1.30 m) originating from beaver gnawing activities, we took diameter only at 15 cm above the ground. Stumps were classified as with or without sprouts (Table 1, Supporting Information 01) based on a visual assessment of the presence/absence of sprouts (1.5 years or more have passed after beaver gnawing) at the time of the field survey or with the fallen stem still connected to the stump; standing tree damages were classified based on the depth of beaver chewing on the trunk, that is, only the bark removal wood and phloem gnawing. Damage severity was assigned based on the presence of vital elements for each gnawed element: for example, the presence of at least one vital shoot from the stump of a felled tree; presence of a vital tree crown for standing trees partially damaged along the stem (Table 1). For all woody stems, we recorded the distance from the riverbank and species. The minimum diameter threshold used for sampling standing trees and stumps was ≥ 3 cm at 15 cm above the ground.

**TABLE 1:** Damage type and related damage severity classes to the single tree stem.

Damage presence	Damage type	Damage severity
No	—	—
Yes	Stump without sprouts	High (felled tree, with no vegetative resprouting)
	Stump with a fallen tree	High (felled tree, with no vegetative resprouting)
	Stump with sprouts	Low (felled tree, with vegetative resprouting)
	Damage to the wood and phloem of a standing tree or shrub	Low (standing living tree)
	Removal of some of the bark of a standing tree or shrub	Low (standing living tree)

### 2.2 ML Models

Three different ML techniques, namely Support Vector Machines (SVM, Boyle 2011), Random Forests (RF, Breiman 2001), and Artificial Neural Networks (ANN, Goodfellow et al. 2016), were intercompared within the context of this study. The three techniques are robust nonparametric learners potentially capable of solving almost any kind of nonlinear problem (Wu and Liang 2018); however, because of the peculiar implementation and according to the No Free Lunch theorem (Wolpert and Macready 1997), each technique can be more or less suitable for a given application. The scope of this intercomparison was therefore to identify, among ANN, RF, and SVM, the ML method more suitable (if any) for the proposed classification.

#### 2.2.1 Support Vector Machines (SVM)

During the training phase of SVM, a quantity of “decision” planes was established to divide the space of input features into subspaces according to a given distance function. The so‐called kernel trick allows mapping the problem into a higher dimensional space where it can be easily solved by linear classification (Vapnik 2000; Boser et al. 1992). However, SVM accuracy was strongly dependent on the hyperparameters' definition. The SVM implemented in this study is based on the Radial Basis Kernel function (RBF) without box constraints (Vapnik 2000). The maximum number of objective evaluation iterations was fixed at 30 after tests carried out with values increasing from 2 to 100. It has been found that a number of iterations smaller than 30 negatively affects the accuracy, while higher numbers increase the computational cost without improving the predictions.

#### 2.2.2 Random Forests (RF)

RF classifiers are also very popular in applications related to earth's system sciences, mainly thanks to their robustness to bias and overfitting (Pal 2005). They can provide very high accuracy with reduced computational cost. RF can be included in the ensemble learning methods (Breiman 2001) since they compute the prediction by averaging the outputs of several independent weak classifiers, running in parallel, which are called decision trees (Quinlan 1993). The RF parametrization includes the number of features in the random subset at each node and the number of decision trees (Camargo et al. 2019). In this study, 50 independent decision trees have been set after tests carried out by iteratively increasing this number from 2 to 500. As for SVM, we found that values smaller than 50 affected negatively the prediction accuracy, while higher numbers increased the computational cost without improving the predictions.

#### 2.2.3 Artificial Neural Networks (ANN)

ANNs are one of the pioneering ML techniques, since their basic element, the perceptron, has been studied since the 50's of the last century, with the aim of replicating the human brain structure (Rosenblatt 1958). Among the various ANN implementations, the feed forward artificial neural networks (FF‐ANNs) have been considered in this study thanks to their capability of solving nonlinear problems (Hornik et al. 1989; Linden and Kindermann 1989). As a main disadvantage, FF‐ANNs are sensitive to outliers: a training representative of all the observable conditions is mandatory for obtaining good generalization capabilities (e.g., Santi 2016). FF‐ANNs are composed of one or more “hidden” layers of perceptrons, plus an input and an output layer. For dimensioning the ANN, the number of neurons and hidden layers must be defined as well as the transfer or “activation” function, which in general is linear, logistic sigmoid, or hyperbolic tangent. The FF‐ANN is trained by the back propagation (BP) learning rule, which attempts to minimize the error between target values and actual output by applying a gradient descent algorithm (Bebis and Georgiopoulos 1994). In this study, the iterative architecture definition technique proposed in Santi et al. (2022) was applied to find the correct dimensioning of the ANN for the proposed problem. Considering the small training data amount, an upper limit to the number of neurons and hidden layers was also implemented to keep the number of bias and weight parameters to compute lower than the training data amount. Moreover, overfitting was controlled by applying the so‐called early stopping rule (Prechelt 1998).

#### 2.2.4 ML Algorithms Implementation and Training

A two‐step ML classification was implemented: in the first step, the trees are classified as damaged or undamaged, while in the second step, the damage severity for the trees in the “damaged” class was further classified as “low” and “high.” Input parameters for all the selected ML algorithms were river ID, tree distance from the riverbank, tree diameter (at 15 cm above the ground, see Crisler and Russell 2010), and tree taxonomic classification at the species level. It is worth mentioning that SVM and RF, being by their nature classifiers, returned directly the 2‐class prediction, while ANN, which is by its nature a regressor, required a further step to partition/separate/subdivide the predictions into binary values: this was obtained by simply rounding to integer the ANN outputs in the range 0–1.

**FIGURE 2:**
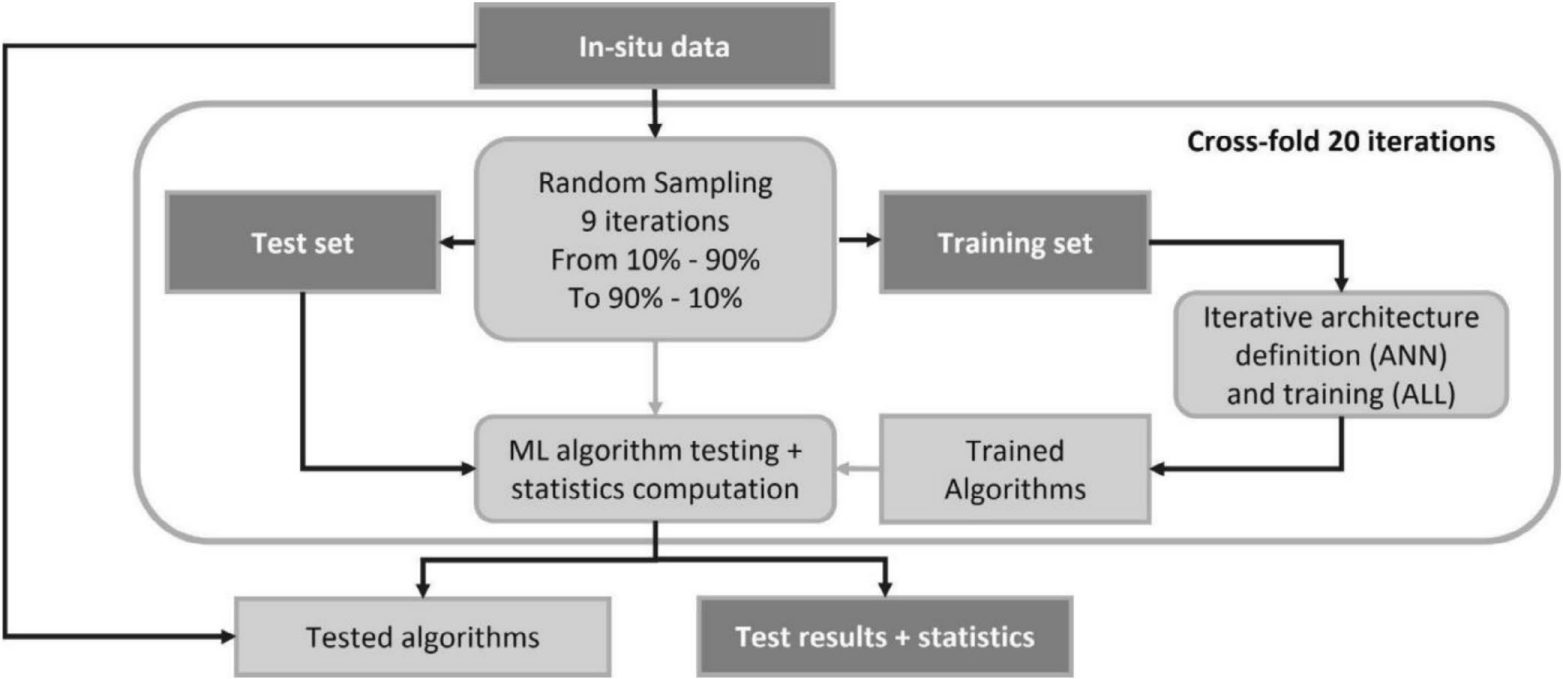
Training and test of the ML algorithms for the first step classification; the datasets are highlighted in dark gray, while the processing steps are highlighted in light gray.

For implementing the first step classification, that is, if the woody stem is with or without damages caused by beaver gnawing activity (Table 1), the three algorithms were trained on subsets of the total dataset randomly sampled and tested on the remaining data. The percentage of the total dataset used for training has been iteratively decreased from 90% to 10% in 10% steps (total nine steps), and consequently, the percentage for testing was increased from 10% to 90%. The amount of data involved in training/testing for each configuration is summarized in Supporting Information 02. This was done to assess the accuracy dependence on the training data amount, given the relatively small dataset available (475 samples) and the general need of large datasets for “robust” training of ML techniques. To reinforce the generalization capabilities of the trained algorithms, a cross‐fold strategy has also been applied, by repeating the entire processing 20 times, including random sampling of training data, training, and test. The results presented here were the average of the 20 iterations. The main steps of the training and testing for the first step classification are summarized in the flowchart of Figure 2.

For implementing the second step classification, which was aimed at separating the damaged trees with “low” or “high” damage severity (Table 1), the ML classifiers were based again on SVM, RF, and ANN with the same input configuration of the first step implementation. In this case, the ML training was carried out using only the conventional 80%–20% combination for training and testing since the small dataset did not allow lowering the percentage of data for training without compromising the classification accuracy. The damaged class was indeed composed of 159 samples out of the total 475, 127 of which were used for training and the remaining 32 for testing. The same 20 cross‐fold strategy used for the first step was applied.

A predictor importance analysis was also implemented to identify, between those sampled in situ, the main parameters capable of influencing the beavers' gnawing activities on riparian woodlands. The analysis was conducted by re‐running the ML algorithm with input subsets randomly combined, according to Breiman (2001) that defined the importance of the predictor *s* based on the difference in prediction error for the model run including *s* compared to the model run without *s*.

**FIGURE 3:**
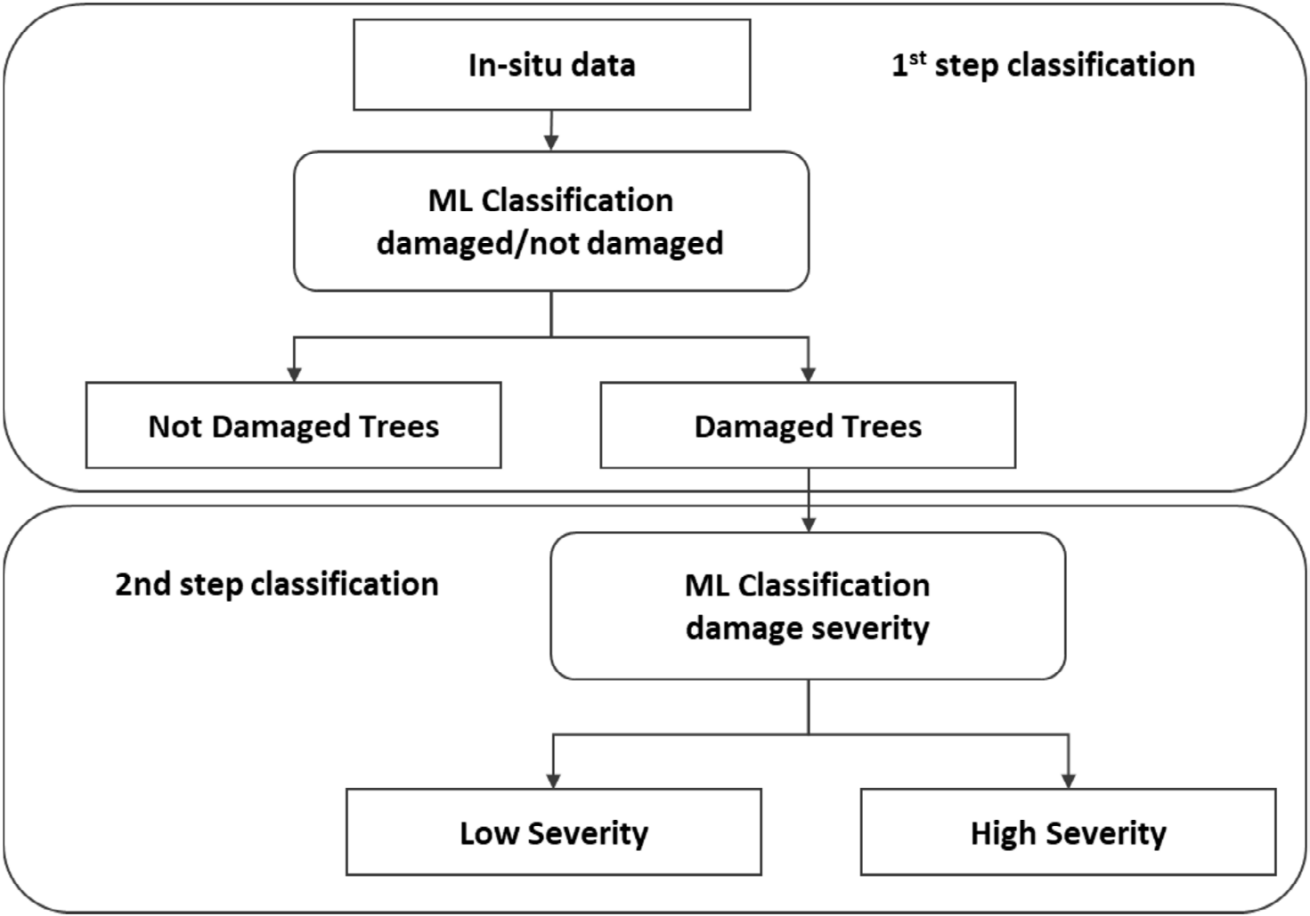
Top‐level scheme of the classification cascade.

The top‐level scheme of the entire process, including both classification steps, is represented in Figure 3.

## 3 Results

### 3.1 Stand Metrics

Main stand structural attributes showed the good representativeness of the selected sample with respect to compositional and structural variation of the Mediterranean riparian ecosystem of Central Italy (Table 2). Based on basal area proportions, riparian woodlands were classified as stands dominated by *Populus nigra* and *Populus alba* ; stands dominated by *Alnus glutinosa* with *P. nigra* as a secondary species; and mixed stands where *P. nigra* co‐occurs with *Salix alba* or *Robinia pseudoacacia* . The latter was primarily observed in the Merse River, reflecting recent logging policies that favored its expansion (Angiolini et al. 2023). Ombrone River stands had lower tree density but higher mean diameter than the other rivers, likely due to differences in water flow and sediment dynamics. Within‐river variability in woody structure was also notable, as indicated by high Quadratic Mean Diameter values, reflecting considerable size heterogeneity within individual stands.

**TABLE 2:** Main stand structural attributes (means among three plots), considering all trees (gnawed and non‐gnawed by beavers).

River	plotID	QMD mean	MD mean	N/ha mean	G/ha mean	First species (G/ha)	Second species (G/ha)
Ombrone	01_O	19.5 (7.7)	13.3 (5.8)	2400 (246.6)	78.3 (50.5)	*Populus nigra* (44%)	*Populus alba* (41%)
	02_O	17.9 (8.8)	13.6 (6.1)	1433.3 (776.7)	30.3 (16.3)	*Populus nigra* (86%)	*Salix alba* (13%)
Tevere	01_T	14.4 (2.5)	12.7 (2.2)	1966.7 (665.8)	30.7 (8.8)	*Alnus glutinosa* (56%)	*Populus nigra* (32%)
	02_T	13.6 (0.7)	12.2 (0.8)	3133.3 (513.2)	45.7 (7.4)	*Populus nigra* (49%)	*Alnus glutinosa* (33%)
Merse	01_M	18.7 (8.1)	15.2 (8.0)	2733.3 (1266.2)	66.1 (28.6)	*Populus nigra* (84%)	*Alnus glutinosa* (10%)
	02_M	12.4 (3)	10.2 (2.3)	2833.3 (1059.9)	32.9 (13.2)	*Populus nigra* (61%)	*Robinia pseudoacacia* (18%)

*Note:* In brackets, standard deviation. The reported tree species are the dominant species in terms of basal area (G/ha).

Abbreviations: G/ha, basal area per ha; MD, mean diameter; N/ha, tree density (number of trees per ha); QMD, quadratic mean diameter.

Beaver activity was concentrated within the first 10 m from the riverbank (Supporting Information 3) for all rivers. Damaged trees ranged up to 32 cm in diameter (measured at 15 cm above ground), but most (91%) were below 19 cm.

### 3.2 Prediction Results

**FIGURE 4:**
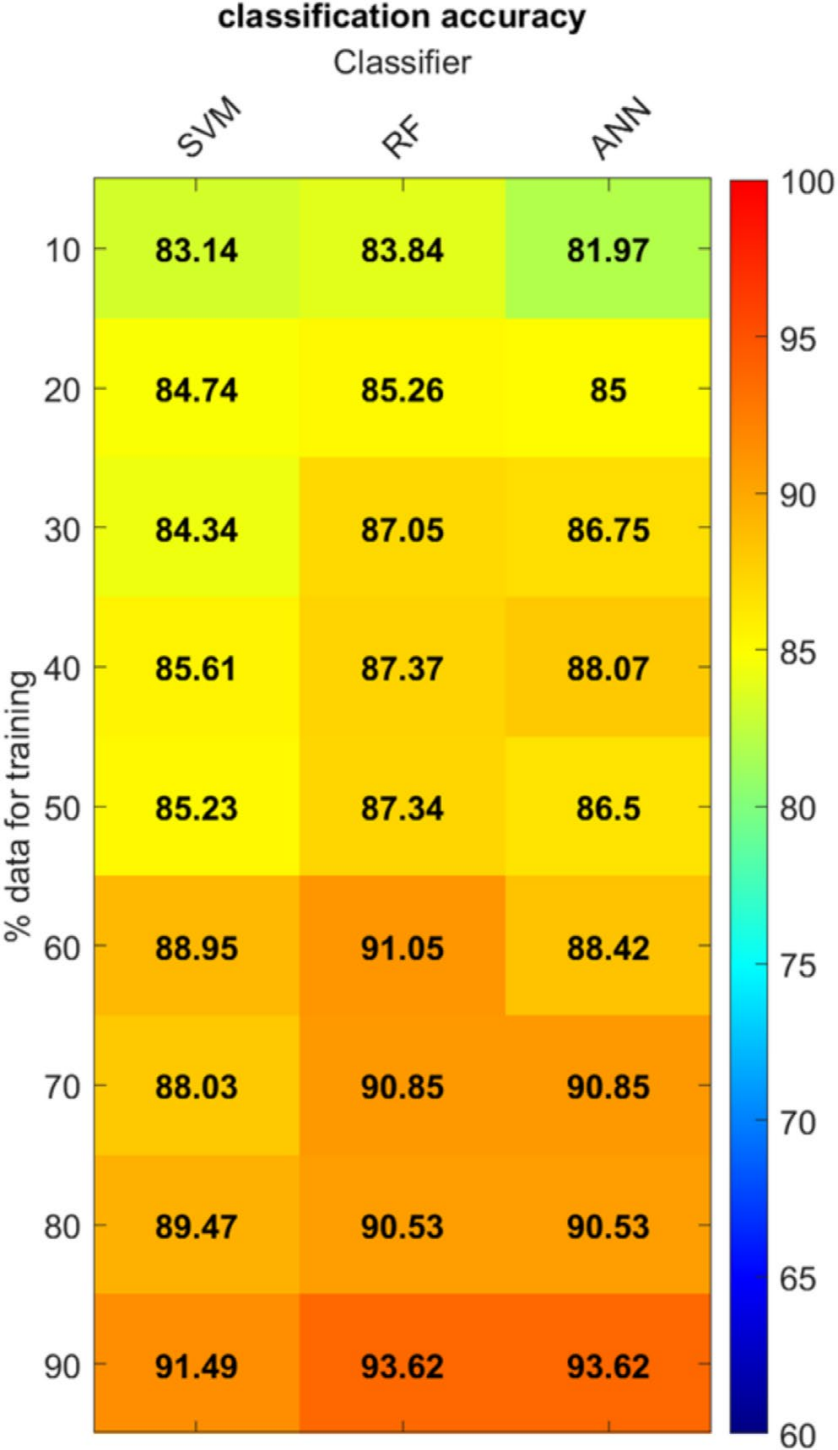
Accuracies obtained by the three ML algorithms in the first step classification (damaged/undamaged) as a function of the % of data used for training. The prediction accuracy color is coded from blue to red for each algorithm and percentage of training data.

The classification accuracies obtained in the first step by the three ML algorithms as a function of the amount of data used for training are summarized in Figure 4. We would stress that the results refer to tests on the subsets not involved in training, which means from 90% of the total dataset if using 10% of data for training to 10% of data if using 90% for training. Accuracies are computed against the “true” reference from in situ measurement, as a ratio between correctly predicted values and total data amount. Overall, ANN and RF obtained the best results, slightly outperforming SVM. As expected, the accuracies of all three algorithms increase when increasing the percentage of data used for training: with the 90%–10% combination, as shown in the last line of the Figure 4, all the algorithms reach accuracy between 90% and 95%, while the accuracies are around 90% for the “canonical” 80%–20%, which is the most widely adopted for ML applications. However, looking at operational applications and consequently at the need to limit the amount of training data, the results obtained by the first three combinations, from 10%–90% to 30%–70%, are even more interesting, since the accuracy is in all cases above 80% and in some cases close to 90%. Focusing, for instance, on the 20%–80% case, it can be observed that 95 data samples are enough to train the ML algorithms for predicting the damaged/undamaged status of the remaining 380 wood samples with an accuracy ≃85%.

**FIGURE 5:**
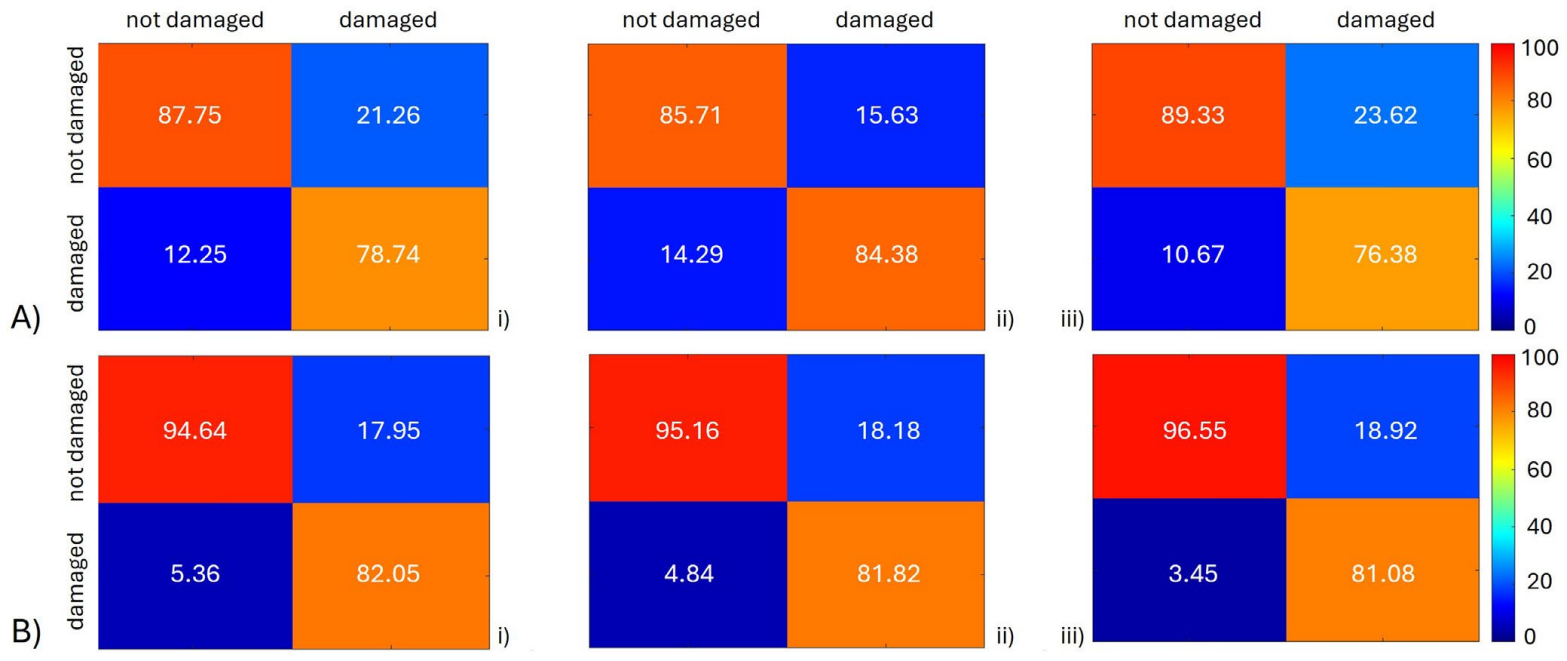
(A) Results of the first step classification (damaged/undamaged) represented as accuracy matrices for SVM (i), RF (ii), and ANN (iii) algorithms trained and tested with the 20%–80% combination; (B) results of the first step classification (damaged/undamaged) represented as accuracy matrices for SVM (i), RF (ii), and ANN (iii) trained and tested with the 80%–20% combination. Target values are in the columns, estimated in the rows.

The accuracy matrices for the combination 20%–80% are shown in Figure 5, A: each matrix shows the percentage of correctly classified, false positives, and false negatives for each of the three ML algorithms, color coded from blue (low values) to red (high values). ANN shows the better result of undamaged classification, by correctly classifying about 90% of the wood samples, while RF obtains the best result in damaged classification, by correctly classifying ≃85% of the damaged wood samples. The % of false positives and false negatives ranges from a minimum of 10% up to a maximum of ≃24%, depending on the ML algorithm.

If considering the specular 80%–20% combination, that means 380 data for training and 95 for test, the result, although improved, can still be considered comparable (Figure 5B): the ANN capability of identifying the undamaged samples increases up to ≃96% but the RF accuracy in identifying the damaged samples remains ≃85% as in the previous case.

**FIGURE 6:**
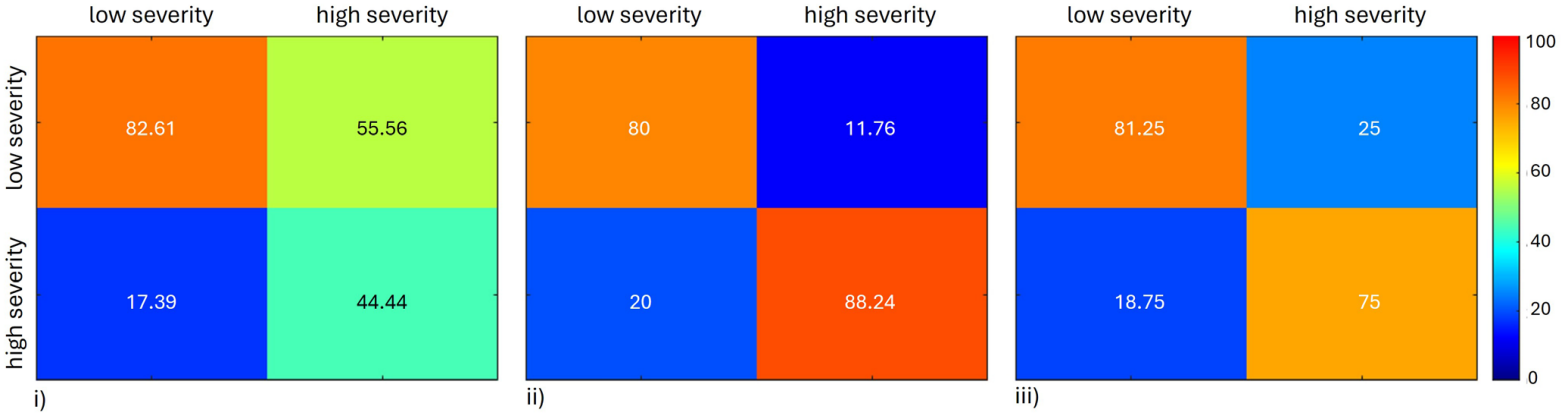
Results of the second step classification (low/high damage severity) expressed as accuracy matrices for SVM (i), RF (ii), and ANN (iii) algorithms trained and tested with the 80%–20% combination. Target values are in the columns, estimated in the rows.

The second step classification into the two damage severity classes resulted in an accuracy *A* = 72% for SVM, *A* = 84% for RF, and *A* = 78% for ANN. These numbers refer to the test set, which is composed of 20% of data not used for training, that is, 32 samples out of the total 159 belonging to the “damaged” class (see Supporting Information 02). The corresponding accuracy matrices are shown in Figure 6.

In detail, RF (Figure 6, ii) outperformed the other two methods by identifying correctly ≃80% of the samples for “low” severity class and ≃88% in the “high” severity class. ANN was able to identify correctly ≃81% of the samples in “low” severity living class but obtained a larger error for the “high” severity class with ≃75% of samples correctly identified. SVM provided similar results to RF for “low” severity class (≃83% samples correctly identified) but was able to identify only ≃44% of samples in Class 2, achieving overall the worst result. In general, the ML algorithms obtained lower accuracy in this “low”/“high” severity class separation than in the damaged/undamaged classification: this can be mainly attributed to the smaller dataset that does not completely represent the space of solutions, the damaged class being composed of 159 samples (72 with low damage severity and 87 with high severity) out of the total 475. Arguably, increasing the training set will also allow improving accuracy.

### 3.3 Predictor Importance Analysis

The predictor importance analysis was based on the RF algorithm, which was the one providing better performance, to identify the main parameters capable of influencing the beavers' gnawing activities on riparian woodlands. In Step 1 classification, the main parameters influencing the beavers' gnawing activities are the distance from the river, which contributed ≃40% to the result, and the tree diameter (≃30% contribution). Tree species and river ID contributed to the overall classification with a percentage below 20% each. In the second step classification (trees with “low” or “high” severity classes), the contribution of the two main parameters remained almost the same, while the tree species weight increased to about 30% and river ID decreased to about 10%.

## 4 Discussion

Rapid assessment of detailed distribution models and vegetation impact quantification of the species would be necessary to implement timely and effective management strategies. Field data collection of wild fauna impacts is very challenging and has plenty of critical issues, from sampling effort feasibility to human disturbance to wild fauna (Witmer 2005; Tuia et al. 2022). Empirical models of beaver impacts on riparian forests have traditionally relied on regression approaches (e.g., Trentanovi et al. 2023; Juhász et al. 2023). Our study demonstrated that machine learning (ML) methods, particularly Artificial Neural Networks (ANN) and Random Forests (RF), provide a viable alternative to enhance model robustness and predictive accuracy (Hamidi et al. 2021). By reducing reliance on extensive field data, ML techniques can streamline the assessment of beaver impacts in riverine systems, while capturing complex, non‐linear interactions among environmental variables (Liu et al. 2018). Consistent with previous research (Mahoney and Stella 2020; Piętka et al. 2023; Wilson et al. 2024), our results indicate that stem distance from the riverbank and stem diameter are the primary predictors of beaver activity. This can be explained by considering the stronger combined effect of tree species and distance in the effort (i.e., time to dedicate to the total or partial trunk gnawing) for feeding activities (see Trentanovi et al. 2023). These factors drive structural changes in riparian stands, such as basal area reduction and increased mean diameter, particularly in intensively impacted areas and in portions of the forest closest to the riverbank (Trentanovi et al. 2025). Conversely, the influence of tree species was limited, likely reflecting the low tree diversity of our surveyed stands and the dominance of Salicaceae species, which is typical of European riparian woodlands (Piton et al. 2020; Juhász et al. 2022). These findings reinforce the notion that structural characteristics, rather than species identity, primarily mediate beaver foraging patterns. Our two‐step ML classification approach proved highly effective. In the first step, the algorithms were able to classify damaged/undamaged trees with an accuracy (*A* % of the samples correctly classified normalized by the total number of samples) that reached a maximum *A* ≃ 94% when considering the 90%–10% train/test ratio. These metrics did not degrade excessively when attempting to reduce the subset of data used for training: by using only 20% of data for training (95 samples only—see Supporting Information 02), all the algorithms obtained a classification accuracy *A* ≃ 85% over the remaining 380 samples (80%) not involved in training. Similar results were obtained in Step 2 (identification of trees with “low” or “high” severity classes among those identified as damaged): in this case, despite the very small dataset (total 159 samples, 127 of which were used for training and 32 for test), the obtained accuracy was about 85%. This result could support a significant reduction of the inventory activities (Pichler and Hartig 2023), which could be limited to the sampling needed for setting up the training set, while the classification of the remaining could be entrusted to the ML algorithms (Besson et al. 2022). The intercomparison of the three ML algorithms also pointed out that RF is the most suitable for this kind of classification in terms of both classification accuracy and computational cost.

However, the proposed approach also has limitations. ML models require careful preprocessing to avoid outliers and ensure balanced class representation, as unbalanced datasets may compromise algorithm training, particularly for ANN (Thessen 2016). In our study, these constraints justified the two‐step classification approach, rather than attempting direct multi‐class prediction. It is worth mentioning indeed that an attempt to classify directly in the three classes of damage intensity (i.e., no damage, lowly damaged, and highly damaged) provided worse results than the two‐step implementation shown here, because of the unbalanced number of samples in the three classes that negatively affected the training convergence of all the ML algorithms. Moreover, the study performs a visual assessment of stumps originated by beaver gnawing activity, without deeper inquiry on the stump vitality with analytical methods. Since no resprouting shoots were observed on the stumps for more than 1.5 years, we classified these tree elements as “highly impacted,” assuming that various environmental factors had reduced their vitality. Typically, stumps of *P. nigra* and *S. alba* cut by beavers in autumn–winter are expected to resprout the following spring (Kuzovkina and Quigley 2005; Fischer et al. 2022). The lack of regrowth may therefore be related to factors such as the intensity of beaver gnawing in the dormant bud zone (Lehtilä 2000), stress conditions like severe drought, or the depletion of non‐structural carbohydrate reserves (Palacio et al. 2014). But we cannot exclude future resprouting of secondary shoots, for example, new root sucker or the regeneration of new basal shoots following other natural events of microclimatic condition changes and thus their influence on future structure and genotypic composition of dominant species (Bailey et al. 2004). Finally, while our analysis clarifies general patterns of beaver selective foraging, it is important to contextualize these impacts within environmental constraints. For instance, water availability in the root zone can modulate the extent of vegetation change induced by beaver activity (Juhász et al. 2023).

## 5 Conclusion

This study demonstrates the potential of machine learning (ML) to support rapid and effective assessment of beaver gnawing impacts on riparian trees. By combining detailed structural measurements with ML approaches, this study provides both methodological insights and ecological understanding that can inform riverine woodland management and conservation strategies under varying intensities of beaver disturbance (Jones et al. 2009; Mikulka et al. 2022). Our results confirm that stem distance from the riverbank and stem diameter are the primary drivers for beaver gnawing preferences, while tree species and river site variability play a lesser role. ML approaches, including ANN, SVM, and RF, reliably predicted both the likelihood and severity of single tree damage, highlighting their value for reducing field survey effort and enabling timely monitoring of beaver activity. Beyond efficiency, ML models offer flexibility: once trained on representative data, they can generalize to other plots and temporal contexts, allowing managers to track vegetation changes over time with minimal additional sampling. These capabilities provide a practical framework for designing adaptive management strategies, prioritizing conservation actions, and mitigating beaver‐induced impacts while optimizing resources. Overall, the integration of ML into forest monitoring represents a promising step toward more precise, scalable, and data‐driven wildlife and habitat management.

## Author Contributions

**Giovanni Trentanovi:** conceptualization (lead), data curation (equal), methodology (equal), supervision (lead), validation (equal), visualization (equal), writing – original draft (lead), writing – review and editing (equal). **Emanuele Santi:** conceptualization (lead), formal analysis (lead), investigation (equal), methodology (equal), software (lead), validation (equal), visualization (lead), writing – original draft (equal), writing – review and editing (equal). **Emiliano Mori:** data curation (equal), funding acquisition (equal), project administration (equal), writing – review and editing (equal). **Andrea Viviano:** data curation (equal), writing – review and editing (equal). **Alessio Giovannelli:** data curation (equal), writing – review and editing (equal). **Maria Laura Traversi:** data curation (equal), writing – review and editing (equal). **Francesco Sarnari:** conceptualization (lead), data curation (equal), supervision (equal), writing – review and editing (equal).

## Funding

Alessio Giovannelli and Emiliano Mori acknowledge the support of the National Biodiversity Future Center (NBFC) to the National Research Council (CNR), funded under the National Recovery and Resilience Plan (NRRP), Mission 4 Component 2 Investment 1.4—Call for tender No. 3138 of 16 December 2021, rectified by Decree no. 3175 of 18 December 2021 of the Italian Ministry of University and Research funded by the European Union—NextGenerationEU; Project code CN_00000033, Concession Decree No. 1034 of 17 June 2022 adopted by the Italian Ministry of University and Research, CUP: C93C22002810006, CUP: B83D21014060006, CUP: B83C22002930006, Project title “National Biodiversity Future Center—NBFC.” E.M., A.V., and G.T. acknowledge the support by Beaver Trust, grant number: 1185451. Open access funding provided by IFAC—SESTO FIORENTINO within the CRUI‐CARE Agreement.

## Conflicts of Interest

The authors declare no conflicts of interest.

## Supporting information


**Data S1:** ece372649‐sup‐0001‐DataS1.xlsx.

**Appendix S1:** ece372649‐sup‐0002‐AppendixS1.docx.

## Data Availability

All the required data is uploaded as Supporting Information.
