# Supplementary material for: Improved Prediction of Eurasian Beaver Gnawing Preferences in Riparian Habitats: A Machine Learning Approach
Source: Ecol Evol. 2025 Dec 17;15(12):e72649. doi: 10.1002/ece3.72649 (PMC12711435; doi:10.1002/ece3.72649)

**Supplementary materials**

**Supplementary material 01**

Classification of damage type based on the visual assessment

**
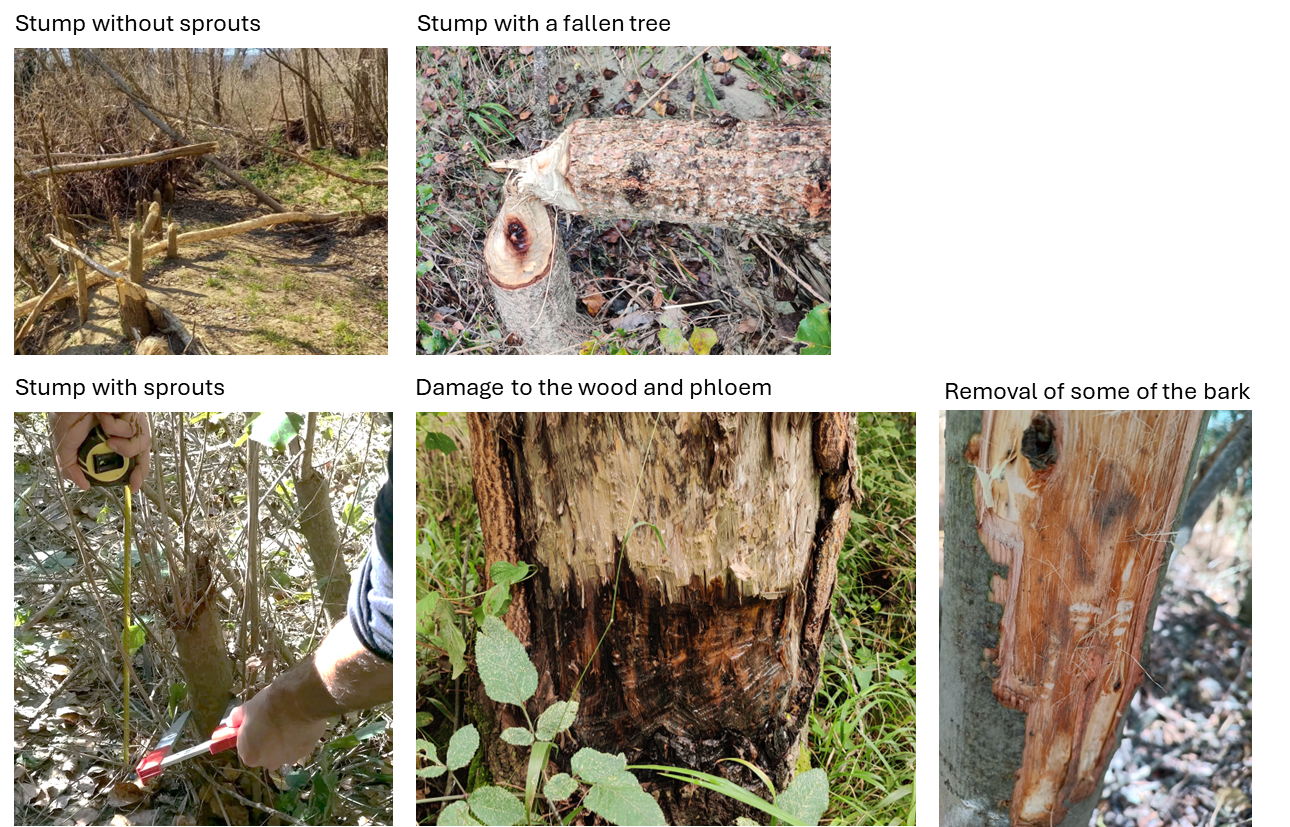
**

**Supplementary material 02**

Amount of data used for training and testing the algorithms for each of the proposed combinations

| **% training-%test** | **# samples for training** | **# samples for test** |
| --- | --- | --- |
| 10-90 | 47 | 428 |
| Step 1 (damaged/not damaged) | | |
| 20-80 | 95 | 380 |
| 30-70 | 142 | 333 |
| 40-60 | 190 | 285 |
| 50-50 | 237 | 238 |
| 60-40 | 285 | 190 |
| 70-30 | 332 | 143 |
| 80-20 | 380 | 95 |
| 90-10 | 427 | 48 |
| Step 2 (living/dead) | | |
| 80-20 | 127 | 32 |

**Supplementary material 03**

Damaged and non-damaged tree distribution by diameter size (DIA) and distance from the riverbank for all surveyed plots.


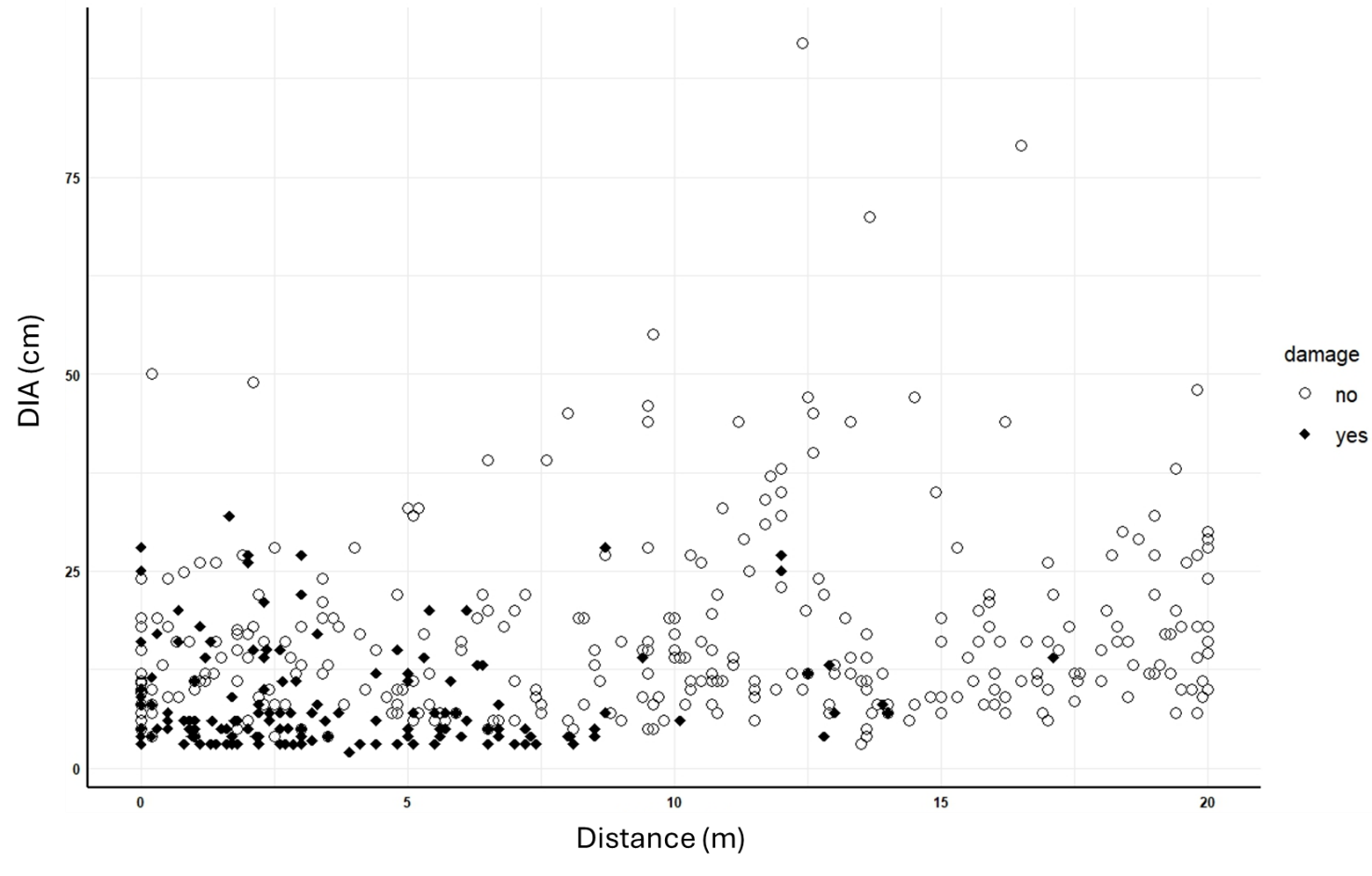

Supplement: Supplementary file 2 — Appendix S1: ece372649‐sup‐0002‐AppendixS1.docx. [file ECE3-15-e72649-s001.docx]
